# Correspondence

**Published:** 1884-01

**Authors:** J. C. Meyer

**Affiliations:** Cincinnati


*To the Editors of the Journal of Comparative Medicine and Surgery:*

GENTLEMEN:—It is with great pleasure that I comply with your request for some notes from my experience in practice during the past year which has been an exceedingly busy one with me, although we have had no eruptions of enzoötic or epizoötic diseases of any importance with the exception of "laryringo-pharyingitis," which appeared almost as frequently as catarrhal complications of the head in horses. Both appear to have more or less connection one with the other, coming as they frequently do, in one and the same individual at the same time. Owners frequently look upon these diseases as having a contagious character, while in my opinion they are simply of an infectious nature. These complications have taken a very mild course with us during the past year which has not always been the case when purpura has appeared as a secondary complication, leading to many fatal terminations. In the above named diseases we often have a yellowish or greenish nasal discharge which gives occasion to the suspicion that glanders may have occurred or is to be suspected. This fear is especially prevalent among horse owners, but is in general unfounded. I am sorry to say that glanders is again becoming unpleasantly frequent among our horses. After the war we had many cases of this disease, but it gradually disappeared. I have seen more cases in the last year than in both the previous years together. Glanders is generally found in the stables of the lower class of horse dealers. If something is not done to prevent this reckless traffic in such horses this disease will continue to extend and be fraught with much danger to our horses, as well as to human beings having contact with them. It would be easy to exterminate this disease, as I am convinced of its strictly contagious character, and it is my opinion that horses in this section of the country are less subject to catarrhal complications than in many others.

We have a law in Ohio which forbids persons dealing in horses afflicted with glanders, as well as using them for work, but the execution of it is left open to any one, and as in everything else, what is everybody's business soon becomes no one's, and the law becomes a dead letter. In such cases as I have met with I have made use of the Society for Protection to Animals, which saves much time, trouble and expense to the veterinary practitioner.

A State veterinarian, with a requisite number of local subordinates, is an absolute necessity in the State of Ohio, as well arranged laws for the suppression of contagio-infectious animal diseases. It seems to me that this end could be easily attained if the profession of the State would unanimously work to bring legislation to bear upon the subject. The newly formed veterinary association of Ohio will probably take this subject up and carry it to some form of completion.

We have at present, as far as I know, no contagious lung disease among our cattle, and it is to be hoped it will long continue so.

The Texas cattle disease appears among our cattle nearly every year, although I have personally seen but two cases during the past one, and have read of its appearing in various localities in the State and causing no inconsiderable loss to cattle owners. The owners of milch cows seem to suffer more than others, but endeavor to keep it quiet, as they fear to lose customers should it become known. There is but little to fear from the sale of such milk, as this function suddenly ceases in the early stages of the disease. These persons endeavor to sell such cows as soon as they observe any symptoms of disease, a procedure which should be prevented by law.

I cannot understand how some European authors can consider this disease to be anthrax (Putz),* nor would it be so had they sufficiently considered the American literature upon the subject. I have subjected the blood and urine to quite an exact examination, and found in the latter numerous hæmatoidin crystals, numberless bacteria, vibrios and epithelial cells, *but no blood cells*, which goes to prove that we have to do with a paranchymotous process in the kidneys; that is, that the blood becomes mixed with the urine in the uriniferous canals of the kidneys. An examination of the blood showed it to be entirely free from bacteria, nothing else abnormal was to be seen the leucocytes not appearing to be numerically increased, by the latter, however, one peculiarity was observed, in that they were frequently to be seen united in twos and threes, surrounded by a common membrane; their protoplasms contained numerous pigment granules.

---

* Or others, as in reality the rinderpest—Gerlach, B.

The disease is not looked upon as contagious nor do we know the exact nature of the infection, though no one doubts its infectious nature.

I have not met with any infection or contagious diseases among sheep, nor have I heard of any. Hog and chicken cholera have acquired but a very slight extension during the past year. I do not know that a case of trichiniasis in human beings has occurred.

It is to be hoped that wherever or whenever either State or local authorities decided to employ veterinarians in the public service that none but graduates of accredited schools will be given such positions.

Wishing you every success,

I am yours, etc.,

J. C. MEYER, SR.

CINCINNATI, NOV. 11, 1883.

